# Flavored Olive Oil as a Preservation Means of Reduced Salt Spanish Style Green Table Olives (cv. Chalkidiki)

**DOI:** 10.3390/foods10020392

**Published:** 2021-02-11

**Authors:** Maria Papapostolou, Fani T. Mantzouridou, Maria Z. Tsimidou

**Affiliations:** 1 Laboratory of Food Chemistry and Technology (LFCT), School of Chemistry, Aristotle University of Thessaloniki (AUTh), 54124 Thessaloniki, Greece; papaposm@chem.auth.gr (M.P.); fmantz@chem.auth.gr (F.T.M.); 2 Natural Products Research Center of Excellence (NatPro-AUTH), Center for Interdisciplinary Research and Innovation (CIRI-AUTH), 57001 Thessaloniki, Greece

**Keywords:** Spanish style table olives, cv. Chalkidiki olives, essential oils, oregano, lemon balm, bay laurel, flavored olive oil, reduced-salt table olive preservation, food safety, response surface methodology

## Abstract

Reformulation of products fermented in brine is a challenging area of research. Continuing the efforts toward the establishment of table olives as a healthy food for all population groups, this study aimed at examining whether olive oil flavored with essential oils can be used as a preservation means for reduced salt Spanish style green table olives (cv. Chalkidiki). Response surface methodology was applied to organize experimentation and assess data. As independent factors, concentrations of the essential oils used (oregano, lemon balm and bay laurel) and time of storage under vacuum were set. Microbiological parameters (pathogens and fermentation-related microbes), color and firmness attributes were used as responses. Models indicated that each essential oil exerted a preservative role to maintain microbiological quality of reduced salt table olives. Concurrently, appearance attributes of the latter were retained at desirable values. Oregano essential oil had a profound role against pathogens. Lemon balm and bay laurel essential oils were found to be important for yeast population control. The results are promising toward the use of flavored olive oil as a preservation means for tailor-made reduced salt table olives, a practice that may enhance local industry innovative activity in a practical and effective way.

## 1. Introduction

Spanish style green table olives are produced in several European and non-European countries following a series of processing steps that involve debittering with dilute alkali solution or simply with water, washing with water to remove alkali excess and then spontaneous or inoculated fermentation in brine for a period of 2–4 months [1]. Bulk storage takes place in fermentation brine. Different lots are then treated and packed in various ways to satisfy innovation efforts of the industry, market demand and consumer expectation for the particular product. Therefore, it is possible to find table olives canned in brine of the same salt concentration as the fermentation one, packed pitted and stuffed with various materials such as almonds, anchovies, peppers and herbs, while there is a tendency to use other packaging materials, such as polyethylene pouches, filled or not with brine or gases (modified atmospheres). In line with nutritional guidelines for salt reduction in the diet [2], the table olive industry is found in a transient period in order to establish this traditional staple food for the habitants of the Mediterranean countries as a healthy product that can be consumed by all groups of the population including children, hypertensive consumers and the elderly. In particular, there is a need to reformulate processing and renovate storage means that include brine with high salt concentrations (4–15% NaCl). Innovative approaches usually employ modified fermentation brines containing less NaCl [3,4,5,6,7,8,9]. However, the data for the shelf life of reduced salt table olives are limited. Regarding Spanish style cv. Chalkidiki green table olives, which have also been registered as a Protected Designation of Origin (PDO) product in Europe [10], Mastralexi et al. [11] pointed out that, although of excellent quality in terms of fruit size, texture, color and microbiological safety attributes, the product suffers from high sodium content. The latter was observed just after fermentation (5% NaCl in edible flesh) and remained high after 12-month storage in the same brine (3.2% NaCl). Sustainability of table olive production in the region of Chalkidiki (Central Makedonia, GR) is very important and faces many challenges the last years (climate change and COVID-19 pandemic) so that local industry is reluctant to introduce innovation in the production line. It is therefore interesting to examine whether alternative solutions can be offered at a post-fermentation stage. 

This study aimed at the production of a tailor-made reduced salt product by: (i) desalting the traditional fermented green table olives by ~25%; and (ii) preserving them in flavored olive oil under vacuum. Flavored oils with essential oils (EOs), herb extracts or the addition of dry herbs are mainly discussed in the literature as end products themselves with reference to their shelf life and sensory characteristics [12,13,14,15]. To our knowledge, this is the first study that examined the possibility to use a flavored oil as a means for table olive preservation due to the antioxidant and antimicrobial properties of its constituents. To serve the aim of the study, the oil chosen as a base was virgin olive oil rich in phenolic compounds that are known to exert high antioxidant activity in vitro and in vivo [16]. Moreover, three EOs (oregano, lemon balm and bay laurel), the flavors of which suit table olives, were selected among an abundance of candidates. According to the literature, the three EOs present differences in their antioxidant and antimicrobial properties due to differences in their chemical composition. Oregano EO is known for the presence of strong antioxidants and antimicrobials such as thymol and carvacrol [17], whereas lemon balm and bay laurel EOs are known for their strong antimicrobial activity due to a variety of non-phenolic terpenoids [18,19]. Response surface methodology (RSM) was applied to study the main and interaction effects of the tested EOs on safety and quality parameters of the reduced salt table olives stored for a period of 12 months.

## 2. Materials and Methods

### 2.1. Samples, Standards and Chemicals

Six plastic kegs (5L) containing Spanish style cv. Chalkidiki green table olives at a olive:brine ratio of 3:2, *w/w* were supplied from the company Athos Olive (Chalkidiki, Greece) (production year 2019). Commercial extra virgin olive oil (EVOO) was used as a base for the flavored ones. The EOs used, oregano, lemon balm and bay laurel, were certified authentic products provided by the industrial partner in the project Aroma Distil (project code: T1EDK-04174). Plant materials (*O. vulgare* ssp. *hirtum*, *M. officinalis*, *L. nobilis*, respectively) were of Greek origin (personal communication). Refined olive oil (ROO) was provided by a local refinery.

Hydroxytyrosol (Htyr) (≥98% purity) was purchased from Extrasynthese S.A. (Genay, France). Tyrosol (Tyr) (98% purity), caffeic acid (CA) (98% purity), phosphoric acid (≥85%) and sulfuric acid (95–98%) were from Sigma-Aldrich (Steinheim, Germany). Acetone (analytical-grade), methanol (HPLC 95%) and *n*-hexane (analytical-grade) were from Merck (Darmstadt, Germany). GC-grade dichloromethane (≥99.9%) was purchased from Honeywell (Charlotte, NC, USA) and *iso*-octane (for spectroscopy) from VWR Chemicals (BDH Prolabo ^®^, Europe). Sodium carbonate anhydrous, sodium sulfate anhydrous, Folin–Ciocalteu (F-C) phenol reagent, acetonitrile (HPLC 95%), hydrochloric acid (37%) and ethanol (analytical-grade) were purchased from ChemLab (Ontario, CA, USA). Methanol (analytical-grade) was from Riedel de Haën (Seelze, Germany) and *d*-tartaric acid from M&B Ltd. (Dagenham, UK). Polyvinylidene fluoride (PVDF) membrane filters (0.22 µm) were obtained from Schleicher & Schuell (Dassel, Germany) and cellulose nitrate membrane filters (0.45 μm) from Sartorius (Goettingen, Germany).

Plate Count Agar (PCA), Potato Dextrose Agar (PDA), Rose Bengal Chloramphenicol Agar (RBCA) base, Violet Red Bile Glucose Agar (VRBGA), Violet Red-Bile Agar (VRBA), de Man–Rogosa–Sharpe (MRS) Agar, Baird-Parker Agar (BPA) Base, and Reinforced Clostridial Medium (RCM) were from Lab M Limited (Heywood, UK) and meet internationally recognized standards. Egg yolk tellurite emulsion and chloramphenicol were from the same company. Ringer’s solution (¼-strength) was from Oxoid Ltd. (Basingstoke, Hampshire, UK). Selenite cystine broth base, USP and Salmonella Shigella (SS) Agar (modified) were purchased from Biolab (Budapest, Hungary). Cycloheximide was obtained from Sigma-Aldrich (Taufkirchen, Germany). Agar was purchased from Duchefa Biochemie (Haarlem, The Netherlands). Ultrahigh-purity water was produced in the laboratory using a Millipore-Milli-Q system.

### 2.2. Desalting Process and Sodium Content Determination

After trial-and-error experiments, a kinetic study for the desalting of the Spanish style green table olives cv. Chalkidiki was carried out in order to achieve sodium reduction in edible flesh by ≥25% and hence to comply with the nutrition claim about “reduced salt” content in the end product [20]. More specifically, 10 olives were immersed in plastic beakers containing tap water (536–581 μS/cm) at a olive:tap water ratio of 1:5, *w/w* and then left at room temperature (T = 25 ± 2 °C) for 12, 24, 48, 72, 96 and 168 h. A 24 h period was found to be sufficient to achieve our goal. For the determination of sodium content, the protocol of Lopez et al. [21] was applied. In brief, 10 olives were manually destoned, ground and homogenized. Four grams (accuracy of ±0.01 g) of olive flesh were weighted in a quartz capsule. The capsule was put in a muffle oven and incinerated at 550 °C for approximately 8–10 h. Ash was dissolved with three parts of 2 mL of 6 N hydrochloric acid solution aided by slightly heating and filtered through a filter paper into a 25 mL volumetric flask. The filter was successively washed up with Milli-Q water, which was also added to the volumetric flask until level. A reagent blank was also prepared. Na was determined in emission mode by a Perkin-Elmer Atomic Absorption Spectrometer PinAAcle 500 (Perkin Elmer, Waltham, MA, USA) with air/acetylene (Air Liquide S.A. Hellas company, Thessaloniki, Greece) oxidizing flame. The optimized instrumental analytical parameters for the determination of sodium were the secondary line for Na (589.4 nm), burner height 5 mm, C_2_H_2_ flow rate 40 L h^−1^, read time 3 s and 3 pixels. All samples were appropriately diluted prior to analysis. Each determination was carried out in triplicate and results were expressed as g per 100 g edible flesh.

### 2.3. CCD of Storage Experiment

An unblocked full central composite design (CCD) of the RSM was constructed to study the main and interaction effects of four factors, namely the percent concentrations of EOs [oregano, (X_1_, % (*w/w*)), lemon balm (X_2_, % (*w/w*)) and bay laurel (X_3_, % (*w/w*))] and storage time (X_4_, months) at five experimental levels coded as −a, −1, 0, +1, +a where −1, 0 and +1 correspond to low, mid and high levels of X_i_, respectively, and a = 5 to establish new extremes for the low and high settings of all factors (Table 1). The low and high levels were selected for each of the above factors and the rest were calculated from the equation shown as a footnote to Table 1. More specifically, EO concentration ranges were 0–1% (*w/w*) for oregano and 0–0.5% *(w/w)* for lemon balm and bay laurel, whereas time span was set from 1 to 12 months. The design consisted of thirty-one experimental runs, set using the software Minitab Release 17.3.1 (free trial, Minitab, Inc., State College, PA, USA). Seven of them were conducted at the center of the design, replicated for the estimation of error. Experiments were randomized to maximize the effect of unexplained variability on the observed responses due to extraneous factors. The effect of the experimental conditions on safety and quality parameters of table olives was examined as described in Section 2.6. The three-dimensional surface plots were also obtained using Minitab Release 17.3.1.

The second-order polynomial model was fitted to the response Y, giving the following equation:

(1) $$Y=\beta_{0}+\beta_{1}X_{1}+\beta_{2}X_{2}+\beta_{3}X_{3}+\beta_{4}X_{4}+\beta_{11}X_{1}^{2}+\beta_{22}X_{2}^{2}+\beta_{33}X_{3}^{2}+\beta_{44}X_{4}^{2}+\beta_{12}X_{1}X_{2}+\beta_{13}X_{1}X_{3}+\beta_{14}X_{1}X_{4}+\beta_{23}X_{2}X_{3}+\beta_{24}X_{2}X_{4}+\beta_{34}X_{3}X_{4}$$

where Y is the dependent variable (response); X_1_, X_2_, X_3_ and X_4_ are the independent variables (factors); and *β*_0_, *β*_1_, …*β*_34_ are the estimated coefficients with *β*_0_ having the role of a scaling constant. Analysis of variance was used to evaluate the quality of the fit of the model to the response by determining the coefficient of determination (*R*^2^), the significance of each parameter through F-test (calculated *p*-value) and the lack-of-fit of the model. The adequacy of fit was also confirmed by various diagnostic tests (normal probability plots, histograms, versus fit plots, versus order plots). Coefficients with a *p*-value < 0.05 were considered significant. The reduced model was obtained through the statistical software by eliminating one at a time insignificant terms and retaining those that supported the hierarchical principle. Multi-response optimization of the fitted polynomials was performed by response optimizer using the same software. 

In addition, two more series of desalted table olives packed in EVOO and ROO with EOs at the middle level (where X_1_, X_2_ and X_3_ equal 0.5%, 0.25% and 0.25% *w/w*, respectively; Table 1) were also stored for 1, 3, 6 and 12 months and used for base comparison effect. One more series in VOO without EOs was prepared and stored under the same conditions and the oil content was used in the UV and FT-IR analyses.

### 2.4. Sample Packaging and Storage

Fifteen different blends of flavored oils consisting of EVOO and the EOs in all combinations presented in Table 1 were prepared. Ten olives were placed in PA/PE pouches (90 µm thickness; oxygen and water vapor permeability of 75 cm^3^ m^−2^ 24 h^−1^ at 23 °C and 75% RH, data provided by the manufacturer) with 20 g of flavored EVOO blend corresponding to each combination of Table 1. The pouches were vacuum-sealed, labeled and stored in the dark at room temperature (T = 25 ± 2 °C) until analysis after 1, 6, 7, 8 and 12 months of storage. All treatment-packages were prepared in triplicate.

### 2.5. Chemical Composition of EOs

A GC-MS analysis was carried out on an Agilent 6890A gas chromatograph equipped with a Mass Selective Detector MSD 5973 mass spectrometer (Agilent Technologies, Palo Alto, CA, USA) and fitted with a DB-WAX capillary column (polyethylene glycol: 30 m × 0.25 mm i.d., 0.33 μm film thickness) (Agilent Technologies, Palo Alto, CA, USA). The gas chromatographic and mass spectrometer conditions adopted are described in a study by Ordoudi et al. [22]. The volatile compounds were identified by comparison of their elution order, available standards and mass spectra with data from the NIST library (Version 2.0f, National Institute of Standards and Technology, Gaithersburg, MD, USA, 2008) and the published literature. 

### 2.6. Tests on Olives

At each sampling point, three pouches (3 × 10 olives) were removed from the storage shelf, the content was mixed and 7 olives were randomly picked and destoned under sterile conditions. Unless otherwise stated, 10 g of flesh were transferred to a stomacher bag with 90 mL of ¼ Ringer’s solution and homogenized at room temperature using a stomacher for 1 min. Samples without or after serial decimal dilutions were plated on appropriate media for microbiological examination. Total viable counts (TVC) were enumerated on PCA after incubation at 25 °C for 48 h; molds were enumerated on PDA (supplemented with 10% tartaric acid) after incubation at 21 °C for 5 d; yeasts were enumerated on RBCA after incubation at 25 °C for 72 h; lactic acid bacteria (LAB) were enumerated on MRS agar after overlay with the same medium and incubation at 30 °C for 72 h. For the enumeration of *Enterobacteriaceae* and coliforms, VRBGA and VRBA were used, respectively (overlay; incubation at 37 °C for 24 h). For the detection of *Staphylococcus*, BPA supplemented with egg yolk tellurite emulsion was used (incubation at 37 °C for 24–48 h). For the detection of *Clostridium*, samples without and after heat treatment (80 °C for 10 min followed by immediate cooling in iced water) were transferred to RCM and incubated at 30 °C for 72 h. For the determination of *Salmonella*, 25 g of olive flesh were transferred to a stomacher bag with 225 mL of selenite cystine broth base and incubated at 37 °C for 18–24 h. Then, the enrichment mixture was plated on SS Agar and incubated at 37 °C for 24 h. The results from plate counts were expressed as log_10_ colony forming units (CFU)/g.

Surface color and the firmness of the olives were assessed according to Mastralexi et al. [11]. Briefly, for color measurements, 10 olives were scanned, using a portable HunterLab-color spectrophotometer (MiniScanTM XE Plus, Reston, VA, USA), at three points per fruit, and the results were expressed as the mean of 30 values. For firmness evaluation, the peel break maximum force (N) was determined from independent measurements on 10 olives with the use of a Texture Analyser (TA.XT2i, Stable Microsystems, Godalming, UK) equipped with a 2 mm diameter cylinder probe. These olives after the above measurements were vacuum-sealed and kept at 4 °C until further analyses.

Olive polyphenols were extracted according to the procedure described by Cabrera-Bañegil et al. [23]. In brief, 10 olives were manually destoned, ground and homogenized. Two grams (accuracy of ±0.01 g) of olive flesh were weighed and extracted once with 10 mL of methanol using an Elmasonic S 30 (H) ultrasound bath (Elma Schmidbauer GmbH, Singen, Germany) for 30 min. The extract was centrifuged at 1700 g (SL 16R Thermo Fisher Scientific, Darmstadt, Germany) at 4 °C for 10 min. The procedure was carried out in triplicate for each sample. Five milliliters of each methanol extract were combined into a representative one, mixed and kept at −18 °C until analysis. The repeatability of the extraction procedure was checked (CV% = 4.9 for TPP, *n* = 5). TPP was estimated using the F-C assay according to Tsimidou et al. [24]. Measurements were carried out at 725 nm after 1 h reaction period. CA was used as an external standard and results were expressed as mg CA/kg edible flesh by means of a calibration curve (100–500 mg/L). The determination was performed in triplicate for each olive extract (CV% ≤5).

Salt content was measured for all CCD samples as described previously (Section 2.2).

The percent moisture content was determined in triplicate; 10 g of olive flesh were weighted in pre weighted aluminum plates and kept at −18 °C overnight to freeze. The samples were then freeze-dried (0.07 mbar, −55 °C) to constant weight. 

### 2.7. Tests in Oils 

Free acidity (expressed as oleic acid percent), peroxide value (PV) and the coefficients of specific extinction at 232 (K_232_) and 270 nm (K_270_) of olive oils used were determined according to the EU Regulation 2568/91 and amendments [25]. The content of Htyr and Tyr was determined by UHPLC according to Tsimidou et al. [24] on a Shimadzu Nexera X2 UHPLC System (Shimadzu Corporation, Kyoto, Japan) equipped with a LC-30AD pump, SIL-30AC autosampler (50 µL loop), a CTO-20AC column oven, a UV-visible diode array SPD-M30A detector and a RF-20AXS fluorescence detector. Repeatability of the extraction of the polar fraction was checked through F-C assay (CV% = 4.7; *n* = 5). Two linear regression curves were constructed, one for Htyr (y_1_ = 2080.2x + 14193, *R*^2^ = 0.999, within 2–40 μg/mL) and one for Tyr (y_2_ = 1371.8 + 2813.1, *R*^2^ = 1.0, within 2–40 μg/mL).

Prior to UV and FT-MIR spectral acquisition, the oil samples were homogenized (30 s) and then equal volumes were combined into a 50 mL falcon tube. The tube was vortexed for 30 s and centrifuged at 10,000 rpm for 5 min. The lipid phase was collected in a new tube. An aliquot of the lipid phase was treated with 10% anhydrous Na₂SO₄, vortexed for 30 s and centrifuged at 10,000 rpm for 5 min. For the UV spectrometric analysis, a Shimadzu UV 1601 spectrophotometer (Shimadzu UV 1601, Kyoto, Japan) equipped with quartz cells (1 cm × 1 cm × 4 cm), was used. The UV spectra of packaged oils were recorded in the region 200–300 nm, after proper dilution in *iso*-octane so that the absorption measurements to comply with the photometric range of the instrument (−0.5 to 3.99). Spectral data acquisition and analysis was done with UVProbe 2.61 (Shimadzu Co., Kyoto, Japan) data handling software. FT-MIR spectroscopic analysis was performed according to Nenadis et al. [26] with a Fourier transform IR spectrometer, IRAffinity-1 spectrometer (Shimadzu Fourier Transform IR Spectrophotometer, Shimadzu Corporation Kyoto Japan) fitted with a Michelson interferometer and a deuterated, L-alanine doped triglycine sulfate (DLaTGS) detector system coupled to an attenuated total reflectance (ATR) accessory with a ZnSe 11 reflection crystal (Specac, Inc., Woodstock, GA, USA). Data preprocessing was carried out using the software IRsolution (version 1.50) supplied by the same manufacturer and included the following actions: “cut” (648–3600 cm^−1^), “smoothing” (10 points), “derivative” (2nd; 10 points) and “arithmetic” (spectra inversion and multiplication by applying a value of −100). The data were extracted, combined and further processed with SIMCA 16.02 software (Umetrics, Sweden). For each spectrum, 598 data points were collected. The FT-IR database of authentic virgin olive oils of LFCT was used as a reference set of spectra. 

### 2.8. Statistical Analysis

Statistical comparisons of the mean values were performed by running an independent *t*-test (*p* < 0.05 confidence level) using the SPSS 25.0 software (SPSS Inc., Chicago, IL, USA). PCA analysis of FT-IR spectra was performed using the SIMCA 16.02 software.

## 3. Results and Discussion

### 3.1. Quality Control of Starting Materials

#### 3.1.1. Table Olives

Table 2 contains the initial values for color and texture parameters as well as TVC, LAB and yeasts population for green table olives cv. Chalkidiki fermented traditionally and those subjected to desalting under the conditions described in Section 2.2 that led to a reduction of ~37% in NaCl (salted olives: 4.8 ± 0.1 g NaCl/100 g edible flesh; desalted olives: 3.0 ± 0.1 g NaCl/100 g edible flesh). TPP content was slightly reduced by this process (~5% loss). 

Statistically significant differences (*p* < 0.05) between salted and desalted olives were observed in color parameters. More specifically, with respect to color, L*, a* and b* values decreased after desalting, a fact that is better illustrated in the calculation of C* and h* values. C* value represents the saturation of the color or otherwise the relationship between intensity and brightness, and its decrease is unwanted because it influences consumer perception of a product appearance [11]. On the other hand, the calculated h* value indicates a yellowish tonality in olives, which is a desirable attribute that remained almost unaffected after desalting. The above findings even for desalted olives are within the range reported in the literature for green table olives of cv. Chalkidiki, using the same instrumentation [9,11]. To our knowledge, there is no published literature supporting that these C* and h* values are unacceptable by consumers. Peel break force was not affected by the desalting process and values were found to be within the range reported in the abovementioned studies. 

Microbiological analyses revealed absence of *Enterobacteriaceae*, coliforms (hygiene indicators) and potentially pathogenic molds and bacteria such as *Staphylococcus*, *Clostridium* and *Salmonella* in both cases. This indicates a safe starting material which complies with the standards of the Codex Alimentarius for table olives [27]. Desalting process exerted a minor effect on the fermentation-related microorganisms. In particular, the levels of LAB and yeasts counts (~7 and 5.5 log_10_ CFU/g, respectively) were in line with those reported in other studies for Spanish style green table olives [11,28]. LAB counted more for the TVC values, being the predominant group in line with previous findings [29].

#### 3.1.2. Oils

The base used for the preparation of the different blends of flavored oil was chosen to be an EVOO that met the legal quality characteristics for free acidity, PV and K_232_ and K_270_ coefficients [25]. Moreover, the EVOO contained a high amount of polar phenolic compounds expressed as total Htyr and Tyr content. The latter are known antioxidants that act as preservatives of virgin olive oil quality upon storage [30] and present specific health properties [16]. The results indicate that the total Htyr and Tyr content was 8.3 mg per 20 g of oil, a concentration higher than the minimum limit of 5 mg/20 g oil above which the olive oil product can bear the health claim on olive oil polyphenols [24]. ROO, devoid of polar phenols, was used for comparison. The analytical data are shown in the Supplementary Materials (Table S1).

#### 3.1.3. EOs 

According to the information provided in the authenticity certificates, the major constituents of oregano EO were thymol (4.22%) and carvacrol (82.90%), whereas those of lemon balm were neral (13.72%), geranial (19.41%) and β-caryophyllene (20.51%). The major constituent of bay laurel EO was 1,8-cineole (49.08%). These data were verified using GC-MS (Figure S1 and Table S2) and were in line with the typical composition of EOs derived from the respective botanical species [18,19,31,32,33,34,35,36].

Summarizing the results from the safety and quality control tests of starting materials, it was concluded that the latter met the requirements in order to proceed to the storage experiment.

### 3.2. Selection of the Conditions for the RSM

As performance of EOs in real food systems may not always be as satisfactory as in in vitro models so that even 100-fold higher concentrations to be required to achieve similar effects [37], the EO concentration ranges should be set so as to exert antimicrobial activity without compromising the sensory profile of the end product. Selection of the total level of addition of EOs to prepare a flavored EVOO was based on relevant literature data [13,38] and minimum inhibitory concentration (MIC) values for LAB [39,40], yeasts [18,41,42] and common pathogens [18,19,35,43,44,45,46,47]. Olfactory thresholds of EOs major volatiles were considered as well [48,49,50]. Oregano, lemon balm and bay laurel EOs were then tested at concentrations up to 1%, 0.5% and 0.5%, *w/w*, respectively. Considering that the production of table olives is annual, storage for one year was deemed sufficient to evaluate critical factors that affect product stability. The amount of 20 g of the oil base was selected by trial-and-error to sufficiently cover the 10 table olives in each pouch. This was considered a precondition for the flavored EVOO to exert its preservative role.

### 3.3. Flavored EVOO as a Means of Preservation

The effect of the three EOs, namely oregano [X_1_, % (*w/w*)], lemon balm [X_2_, % (*w/w*)] and bay laurel [X_3_, % (*w/w*)], as well as of time [X_4_, months], on a series of safety and quality parameters of table olives was assessed for CCD and other storage experiments. The responses shown in Table 3 are discussed in Section 3.3.1, Section 3.3.2 and Section 3.3.3. The second-order polynomial model was fitted to the experimental data for *Staphylococcus* (Y_S_), LAB (Y_L_), yeasts (Y_Y_), C* (Y_C*_), h* (Y_h*_) and firmness (Y_F_) responses, and the simplified equations (Equation (2)–(11)) for Models 1–5 are given in Table 4. ANOVA of Models 1–5 revealed significance of regression (*p* < 0.05) and non-significant lack-of-fit (*p* > 0.05), while coefficient of determination (*R*^2^) values were from 0.539–0.642, which indicate that more than 54% of the variability of the responses is explained by the models. The presence of EOs in the flavored olive oils over the storage period of one year was checked by UV spectra (Figure S2). PCA analysis of FT-MIR spectra of flavored VOOs vs. those of a reference data set for VOOs confirmed UV observations (Figure S3).

#### 3.3.1. Effect on Common Pathogens

Enterobacteria, coliforms, *Salmonella* spp., *Clostridium* spp. and molds were not detected (<10 CFU/g) over one year of storage experiment.

In Months 6 and 7, observation of black colonies on BPA allowed for the detection of *Staphylococcus* spp. (<10^3^ CFU/g) at levels below the upper limit reported in the literature for *S. aureus* [51,52]. As shown in Equation (2), only X_1_ and X_2_ assigned, respectively, to concentrations of oregano and lemon balm EO, had a negative and significant linear effect (at 95% confidence level) with oregano EO bearing the highest coefficient (−66.8). Quadratic terms of X_1_ and X_2_, however, were found to have an opposite effect (*p* < 0.05), which points out that the increase in EOs concentration up to the highest tested values (1.0% and 0.5%, *w/w*, respectively) does not further enhance the negative effect on *Staphylococcus* population. According to the literature, MIC values for *S. aureus* range 0.05–2.9 mg/mL for oregano EO [45,47,53,54,55,56] and 0.2–2.7 mg/mL for lemon balm EO [35,57]. These values are in line with the most effective concentrations found in the present study, i.e. 0.5% (4.6 mg/mL oil) and 0.25% (2.3 mg/mL oil), respectively. Both linear and quadratic terms of bay laurel EO (X_3_) and time (X_4_) and all possible interactions between the examined factors (X_3_X_4_, X_1_X_3_, X_2_X_3_, X_2_X_4_, X_1_X_4_ and X_1_X_2_) showed non-significant effects on Y_S_ response. 

**Figure 1:**
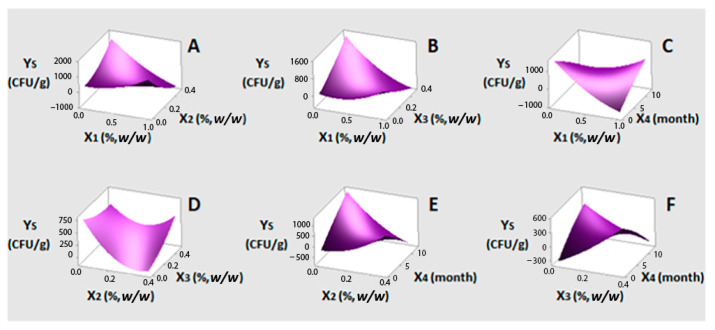
(**A**–**F**) three-dimensional surface plots for *Staphylococcus* spp. growth (Y_S_, CFU/g) as a function of oregano (X_1_%, *w/w*), lemon balm (X_2_%, *w/w*), bay laurel (X_3_%, *w/w*) EOs contents and time (X_4_, month). In all cases, the rest factors were kept constant at their middle levels (0.5%, 0.25% and 0.25% *w/w* and seven months, respectively).

Visualization of the relationships between dependent (Y_S_) and independent factors (X_1_, X_2_, X_3_ and X_4_) is provided through the response surface plots (Figure 1A–F), which express the fitted polynomial equation (Equation (3)). In Figure 1A, where the most significant factors are displayed, it becomes obvious that, in the absence of lemon balm EO, the inhibitory activity of oregano EO starts to decline with increasing concentration. However, setting lemon balm EO at its maximum level (0.5%, *w/w*), oregano EO inhibitory activity was slightly enhanced. Considering that synergism between carvacrol and citral has been previously reported against two *Listeria* spp. [58], a sort of synergism between oregano and lemon balm EOs cannot be precluded. Bay laurel EO exerted a similar effect with that of lemon balm EO in relation to oregano EO (Figure 1B). By keeping oregano EO at middle level and in the absence of bay laurel EO, lemon balm EO exerted a clear inhibitory activity against the pathogen, with increasing concentration (Figure 1D). The co-presence of lemon balm and bay laurel EOs at their highest concentrations showed opposite effects. Such behavior was characterized as antagonistic activity in the case of galangal EO (rich in 1,8-cineole) vs. lemongrass EO (rich in citral) against *S. aureus* [59]. Concerning EOs activity as a function of time (Figure 1C,E,F), all three, and especially lemon balm EO, were found to be effective from the beginning of storage. From the three EOs tested, bay laurel was found to be the least effective.

No pathogens were detected in the two additional series of samples with flavored EVOO or ROO over the 12-month storage period, indicating that EVOO might have simply played the role of EO carrier.

#### 3.3.2. Effect on Fermentation-Related Microorganisms

TVC, LAB and yeasts were all detected and enumerated in table olives. Fluctuation in the populations of all three microbial groups was noted in all samples throughout the storage period. In general terms, TVC ranged 4.6–7.4 log_10_ CFU/g, LAB counts ranged 5.7–7.3 log_10_ CFU/g and yeasts 4.4–5.1 log_10_ CFU/g. 

ANOVA for Y_L_ showed an insignificant lack-of-fit (*p* > 0.05). The regression model was also found insignificant, implying that the changes observed under the conditions tested cannot be plainly assigned to EOs activity. This finding was considered promising, as the dominance of LAB in the final product is desirable to control other spoilage or pathogenic microorganisms. For Y_Y_ (Model 2), only the quadratic terms of X_2_ and X_3_, that is lemon balm and bay laurel EOs, respectively, were found to negatively and significantly (*p* < 0.05) influence yeasts counts with X_2_^2^ exerting the strongest impact (−0.01521) (Equation (4)). Although X_2_ and X_3_ showed no significant linear effects, these factors were included in Equations (4) and (5) as their quadratic effects were significant. Linear X_2_ and X_3_ terms seem to affect the population of yeasts in positive and negative ways, respectively. The desirable effect of X_3_ is enhanced at concentrations higher than the middle level (0.25%, *w/w*). Both linear and quadratic terms of oregano EO (X_1_) and time (X_4_), as well as all possible interactions between the examined factors, showed non-significant effects on Y_Y_. 

**Figure 2:**
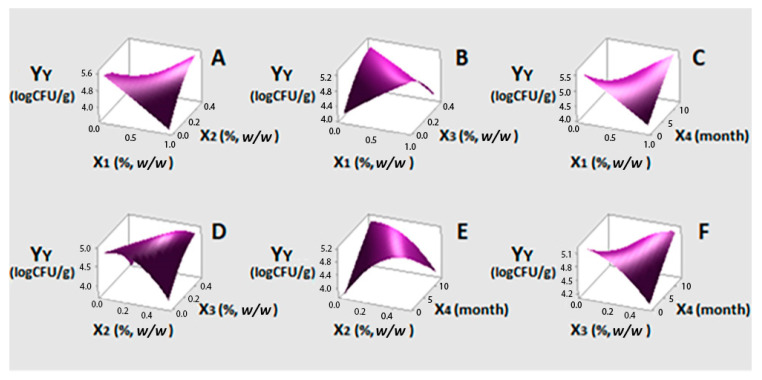
(**A**–**F**) three-dimensional surface plots for yeasts growth (Y_Y_, log_10_ CFU/g) as a function of oregano (X_1_%, *w/w*), lemon balm (X_2_%, *w/w*), bay laurel (X_3_%, *w/w*) EOs contents and time (X_4_, month). In all cases, the rest factors were kept constant at their middle levels (0.5%, 0.25% and 0.25% *w/w* and seven months, respectively).

Response surfaces of Y_Y_ corresponding to Equation (5) are presented in Figure 2. Figure 2D displays the most significant factors. As can be observed, in the absence of bay laurel EO, yeast population drops with the increase in lemon balm EO concentration. In the absence of the latter, yeasts are inhibited due to the increase in bay laurel EO concentration. Co-presence of both EOs was not effective. No relevant literature was found to support this finding. The only information available concerned synergism between galangal (rich in 1,8-cineole) and lemongrass (rich in citral) EOs, at all volume ratios tested, against *Candida albicans* [59]. Oregano in the presence of lemon balm (Figure 2A) or bay laurel EOs (Figure 2B) at all three at their maximum concentrations presented opposite trends that were not found statistically significant and are not discussed further. Nevertheless, it should be mentioned that synergistic effects have been reported in the literature for binary mixtures of pure carvacrol vs. citral and carvacrol vs. 1,8-cineole in model systems against yeast species [60,61]. Concerning EOs activity as a function of time (Figure 2C,E,F), all three were found to be effective from the beginning of storage. The use of ROO instead of EVOO was not found to affect the evolution of LAB and yeast population. LAB population ranged 5.9–6.8 and 5.7–7.0 log_10_ CFU/g and that of yeasts 4.6–5.0 and 4.9–5.1 log_10_ CFU/g, respectively. This is promising for the use of less expensive oils as carriers.

#### 3.3.3. Effect on Color and Texture Parameters

The results of statistical analysis for Y_C*_ (Model 3) showed that linear X_1_ and both linear and quadratic X_4_ had a positive and significant effect at 95% confidence level. The linear term time (X_4_) presented the highest coefficient (0.2947) (Equation (6)). Quadratic term of oregano EO (X_1_), both linear and quadratic terms of lemon balm (X_2_) and bay laurel (X_3_) EOs and all possible interactions between the examined factors showed non-significant effects on Y_C*_ response. The results of statistical analysis for Y_h*_ (Model 4) showed that linear X_1_ and quadratic X_4_ have positive and significant contributions to this color parameter, similarly to what was found for C*. However, there was a significant interaction effect between these two factors (X_1_X_4_), with the strongest, negative impact (−0.499) (Equation (8)). Bay laurel EO (X_3_) was also found to have a negative and significant influence. Linear X_4_ term, although non-significant, was included in Equations (8) and (9) as its quadratic effect was significant. Both linear and quadratic terms of lemon balm EO (X_2_), quadratic terms of oregano (X_1_) and bay laurel (X_3_) EOs and X_3_X_4_, X_1_X_3_, X_2_X_3_, X_2_X_4_ and X_1_X_2_ interactions, showed non-significant effects on Y_h*_ response. 

**Figure 3:**
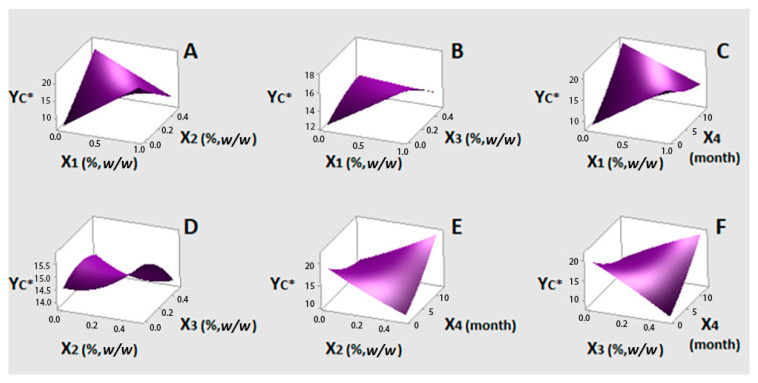
(**A**–**F**) three-dimensional surface plots for color parameter C* (Y_C*_) as a function of oregano (X_1_%, *w/w*), lemon balm (X_2_%, *w/w*), bay laurel (X_3_%, *w/w*) EOs contents and time (X_4_, month). In all cases, the rest factors were kept constant at their middle levels (0.5%, 0.25% and 0.25% *w/w* and seven months, respectively).

**Figure 4:**
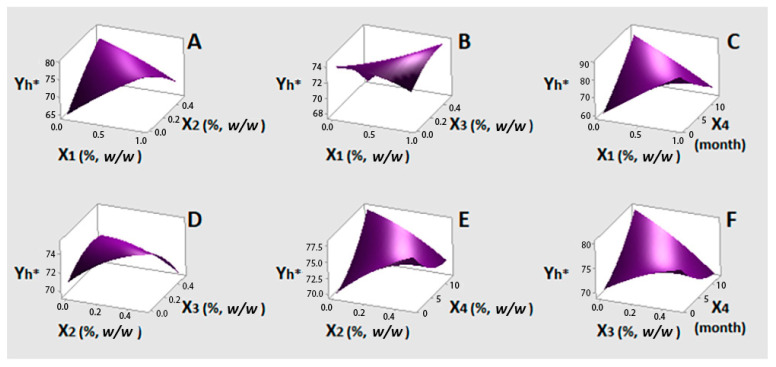
(**A**–**F**) three-dimensional surface plots for color parameter h* (Y_h*_) as a function of oregano (X_1_%, *w/w*), lemon balm (X_2_%, *w/w*), bay laurel (X_3_%, *w/w*) EOs contents and time (X_4_, month). In all cases, the rest factors were kept constant at their middle levels (0.5%, 0.25% and 0.25% *w/w* and seven months, respectively).

Figure 3 illustrates response surface plots for Y_C*,_ which correspond to Equation (7) (Table 4). It is important to observe that the color intensity of olives was retained near its initial value (~16) until the end of the storage period (Figure 3C,E,F). Oregano EO at low levels also contributes to this phenomenon (Figure 3A–C). Response surfaces of Y_h*_ are based on Equation (9) and are presented in Figure 4. Similarly to what was found for C*, low oregano EO content in Figure 3C exerts a positive impact on h*, i.e., Y_h*_ values are close to the initial one (~80). It can be said that the yellowish tonalities were kept rather stable in the presence of this EO. This effect was more pronounced up to six months of storage. Findings for C* and h* changes are in line with trends reported for both of them by Mastralexi et al. [11] during storage of Spanish style cv. Chalkidiki green table olives in brine for 12 months, using the same instrumentation. On the other hand, it can be seen that, by decreasing or even eliminating oregano EO (X_1_), low levels of bay laurel EO (X_3_) (< 0.25%) resulted in some reduction of h* values (Figure 3B,F). In Figure 3E, low lemon balm EO content seems to have a positive impact on h* over time, however the effect is non-significant. In addition, the effect of oregano EO or bay laurel was not changed significantly in relation to the level of lemon balm EO (Figure 3C,D). Overall, of the three EOs, oregano was found to influence color parameter changes less over time. 

With respect to firmness, the statistical analysis for Y_F_ showed that quadratic X_3_ and interactions of X_2_X_3_ and X_2_X_4_ had a positive and significant effect (at 95% confidence level) on firmness with the last one bearing the highest coefficient (0.325) (Equation (10)). On the other hand, interactions X_1_X_4_ and X_3_X_4_ had negative and significant effects on Y_F_ with X_3_X_4_ bearing the highest coefficient (−0.391). Linear terms of all four factors (X_1_–X_4_), although non-significant, were included in Equation (10) and (11) as their interaction effects were found to be significant. Quadratic terms of X_1_, X_2_ and X_4_ and interactions X_1_X_3_ and X_1_X_2_ showed non-significant effects on Y_F_.

**Figure 5:**
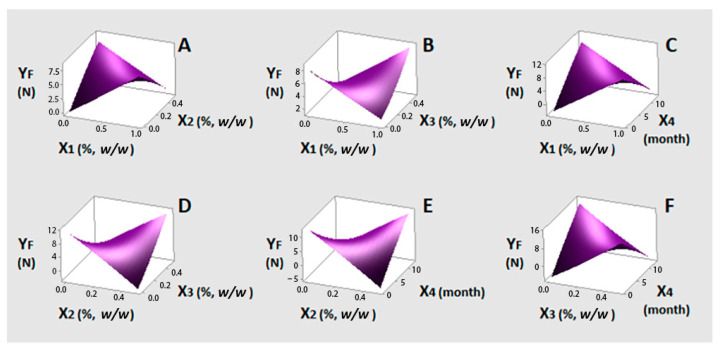
(**A**–**F**) three-dimensional surface plots for firmness (Y_F_, N) as a function of oregano (X_1_%, *w/w*), lemon balm (X_2_%, *w/w*), bay laurel (X_3_%, *w/w*) EOs contents and time (X_4_, month). In all cases, the rest factors were kept constant at their middle levels (0.5%, 0.25% and 0.25% *w/w* and seven months, respectively).

Figure 5 displays the response surface plots for Y_F_, which express the fitted polynomial equation (Equation (11)). Considering Figure 5A–F, two main points can be discussed. Regarding interactions between the EOs, bay laurel EO (X_3_) combined with either oregano (Figure 5B) or lemon balm (Figure 5D) EO at their maximum levels (1% and 0.5%, respectively) improves the firmness of table olives with increasing content. This can be attributed to the fact that bay laurel EO at high levels (0.5%) was one of the two significant factors that inhibited yeast growth. It has been reported that some olive-related yeast strains can cause softening of the fruit due to the production of various extracellular enzymes [62]. As shown in Figure 5C,E,F, low levels of oregano (Figure 5C) and bay laurel (Figure 5F) EOs seem not to affect response (Y_F_) with time (X_4_). Lemon balm on the other hand (Figure 5E) has the opposite effect even from the beginning of the experiment. Overall, the presence of all three EOs, and especially that of bay laurel, can be considered advantageous, as they manage to make time (X_4_) a non-significant factor for Y_F_ response. 

#### 3.3.4. Effects on Nutritional Parameters

The TPP content of table olives was not drastically influenced under the conditions tested. Indeed, for the total of 31 runs assessed, the calculated %TPP changes were found to be within the range of analytical error (approximately ± 5%) for 12 cases. In 16 cases, the %TPP loss observed ranged 8.5–22, whereas, in two samples, a loss around 30% was noted. An increase of ~7% in TPP content was observed in one sample only (Run 27, Table 1), which is probably related to oregano EO phenolic constituents that were at their maximum concentration (X_1_, 1%). 

Salt content remained almost intact during the storage experiment, as expected, in comparison to the initial value of the desalted olives. 

### 3.4. Overall Discussion and Future Perspectives

**Figure 6:**
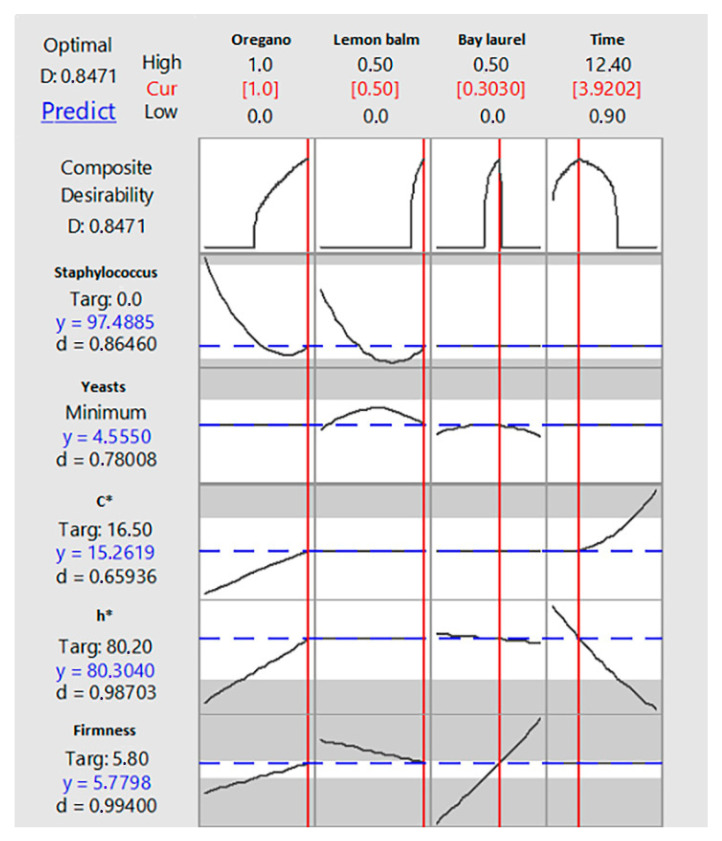
Multiply response optimization plot for growth of *Staphylococcus* (Y_S_) and yeasts (Y_Y_), C* (Y_C*_), h* (Y_h*_) and firmness (Y_F_).

Values of independent variables (X_1_, X_2_, X_3_ and X_4_) determined to be optimum for Y_S_, Y_Y_, Y_C*_, Y_h*_ and Y_F_ responses by RSM optimization approach are presented in Figure 6. The respective oregano, lemon balm and bay laurel EOs content and storage time combinations of 1.0%, 0.5% and 0.3%, respectively, and 3.9 months were predicted as the optimum values to simultaneously: (a) reach the target zero value for *Staphylococcus* population (Y_S_); (b) minimize yeast population (Y_Y_) at an acceptable level that cannot affect organoleptic acceptance; and (c) retain the color (Y_C*_ and Y_h*_) and firmness (Y_F_) of table olives at values observed just after desalting. The predicted values for the responses seem encouraging toward the direction of the production of tailor-made reduced salt table olives preserved under mild conditions, although this product has a shorter shelf life than the conventional one to avoid potential risk from *S. aureus*. Occurrence of *S. aureus* in commercial table olives with typical salt content (~ 5% or more) has been previously reported at low levels (<100 CFU/g or CFU/mL) [27,63,64] possibly due to its ability to grow at water activity values as low as 0.83 [65]. In this study, salt content of the olives used was reduced to around 3% (*w/w*) a fact that may favor *S. aureus* growth more.

The present findings open a path in the local industry of green table olives to examine the use of flavored oils as an effective preservation means of tailor-made products. Such a practice may enhance innovative activities in the medium size local enterprises in a practical and effective way providing that consumer safety and acceptability are ensured. The latter issues will be addressed in further studies. 

## Figures and Tables

**Table 1:** Experimental design for four-factor five-level CCD.

**Factor**			**Variable**		**Levels**				
					**Coded Values ^1^**				
					**−a**	**−1**	**0**	**+1**	**+a**
					**Actual Values**				
oregano (%, *w/w*)			X_1_		0	0.4	0.5	0.6	1
lemon balm (%, *w/w*)			X_2_		0	0.2	0.25	0.3	0.5
bay laurel (%, *w/w*)			X_3_		0	0.2	0.25	0.3	0.5
time (month)			X_4_		0.9	5.5	6.65	7.8	12.4
**Run**	**Factor**				**Run**	**Factor**			
	**X_1_**	**X_2_**	**X_3_**	**X_4_**		**X_1_**	**X_2_**	**X_3_**	**X_4_**
1	0.5	0.25	0.25	0.9	17	0	0.25	0.25	6.65
2	0.5	0.25	0.25	6.65	18	0.5	0.25	0.25	6.65
3	0.5	0.25	0.25	6.65	19	0.4	0.2	0.3	7.8
4	0.6	0.3	0.2	7.8	20	0.5	0.25	0.25	6.65
5	0.5	0.25	0.25	6.65	21	0.5	0	0.25	6.65
6	0.5	0.25	0.25	6.65	22	0.4	0.2	0.2	5.5
7	0.5	0.25	0	6.65	23	0.5	0.5	0.25	6.65
8	0.6	0.3	0.3	5.5	24	0.4	0.3	0.3	5.5
9	0.5	0.25	0.25	12.4	25	0.4	0.2	0.3	5.5
10	0.4	0.2	0.2	7.8	26	0.4	0.3	0.3	7.8
11	0.6	0.3	0.3	7.8	27	1	0.25	0.25	6.65
12	0.4	0.3	0.2	7.8	28	0.5	0.25	0.5	6.65
13	0.6	0.2	0.3	7.8	29	0.6	0.2	0.2	7.8
14	0.6	0.3	0.2	5.5	30	0.5	0.25	0.25	6.65
15	0.6	0.2	0.2	5.5	31	0.4	0.3	0.2	5.5
16	0.6	0.2	0.3	5.5					

^1^ $\;\mathrm{Coded}\;\mathrm{value}=\frac{\mathrm{actual}\;\mathrm{level}-\left(\mathrm{high}\;\mathrm{level}+\mathrm{low}\;\mathrm{level}\right)/2}{\left(\mathrm{high}\;\mathrm{level}-\mathrm{low}\;\mathrm{level}\right)/2}.$

**Table 2:** Physical and microbiological parameters ^1^ of starting materials (table olives).

**Table Olives**	**Physical Parameters**					
	**Color**					**Firmness**
	L* ^2^	a* ^2^	b* ^2^	C* ^2^	h* ^2^	**Peel break force (N)**
Salted	46.3 ± 1.4 ^a^	3.8 ± 0.6 ^a^	24.6 ± 2.1 ^a^	24.9 ± 2.1 ^a^	81.1 ± 1.2 ^a^	5.4 ± 1.4 ^a^
Desalted	33.9 ± 1.4 ^b^	2.8 ± 0.4 ^b^	16.2 ± 2.1 ^b^	16.5 ± 2.1 ^b^	80.2 ± 1.2 ^b^	5.8 ± 1.8 ^a^
	**Microbiological Parameters**					
	**TVC** **log_10_ CFU/g**		**LAB** **log_10_ CFU/g**		**Yeasts** **log_10_ CFU/g**	
Salted	7.1 ± 0.1 ^a^		6.8 ± 0.3 ^a^		5.0 ± 0.1 ^a^	
Desalted	7.1 ± 0.3 ^a^		6.6 ± 0.0 ^a^		5.4 ± 0.1 ^b^	

^1^ Results are given as the mean value ± SD (*n* = 3; *n* = 30 for color; *n* = 10 for firmness). Values within the same column bearing different lowercase letters as superscripts are statistically different (*p* < 0.05); ^2^ L*, a*, b* = CIE rectangular coordinates (lightness, coordinate red/green, coordinate yellow/blue, respectively). $C^{*}=\sqrt{{\left(a^{*}\right)}^{2}+{\left(b^{*}\right)}^{2}}$, $h^{*}=\tan^{-1}\left(\frac{b^{*}}{a^{*}}\right)$.

**Table 3:** Experimental response values for growth of *Staphylococcus* (Y_S_), LAB (Y_L_) and yeasts (Y_Y_), color (Y_C*_ and Y_h*_) and firmness (Y_F_).

Run	Response ^1^					
	Y_S_ (CFU/g)	Y_L_ (log_10_ CFU/g)	Y_Y_ (log_10_ CFU/g)	Y_C*_	Y_h*_	Y_F_ (N)
1	0	6.9	4.9	14.5	74.4	3.8
2	3.3	6.2	5	14.5	73.0	4.6
3	3.3	6.3	5.1	14.6	74.3	4.1
4	0	7.1	4.9	15.8	73.8	4.7
5	200	6.7	5	14.2	73.0	4.0
6	110	6.8	4.9	14.7	73.6	3.8
7	63.3	6.7	4.7	14.8	73.7	4.9
8	10	6.0	4.8	14.5	74.6	5.4
9	0	5.7	4.9	16.8	75.5	3.8
10	0	6.4	4.9	13.9	73.2	4.2
11	0	7.2	4.9	15.3	72.1	3.7
12	45	7.0	4.7	15.3	74.4	4.9
13	0	7.1	4.9	15.6	73.5	3.6
14	30	6.8	4.8	14.6	74.6	2.4
15	0	6.2	4.8	15.7	72.9	5.2
16	10	6.4	4.7	14.9	74.6	5.1
17	720	6.9	4.9	14.1	71.8	4.1
18	6.7	6.6	4.7	15.7	74.9	4.7
19	50	7.0	5	15.0	72.9	3.6
20	3.3	6.2	5	14.4	72.6	4.3
21	706.7	6.9	4.4	14.9	72.6	4.0
22	10	6.6	4.9	13.9	72.9	4.2
23	10	6.2	4.6	15.2	73.0	3.8
24	440	5.8	4.8	13.6	72.4	4.1
25	73.3	5.9	4.8	12.9	71.4	3.7
26	60	7.3	4.8	15.4	74.3	4.2
27	0	6.7	4.9	15.3	73.9	4.5
28	203.3	6.3	4.7	13.9	71.7	4.9
29	0	6.7	5	15.4	74.5	4.1
30	350	6.8	4.8	14.2	73.4	4.4
31	183.3	6.5	4.8	14.0	72.9	3.0

^1^ Results are given as mean values (*n* = 3 for microorganisms; *n* = 30 for color parameters; *n* = 10 for firmness).

**Table 4:** Model equations for Y_S_, Y_Y_, Y_C*_, Y_h*_ and Y_F_ responses.

Model	Polynomial Equation ^1^	
	Coded Value of Factors	Actual Value of Factors
1	$Y_{S}=55.7-66.8X_{1}-43.3X_{2}+11.88X_{1}^{2}+11.82X_{2}^{2}$ (2)	$Y_{S}=1199-1857X_{1}-3230X_{2}+1188X_{1}^{2}+4727X_{2}^{2}$ (3)
2	$Y_{Y}=4.9022+0.0094X_{2}-0.0051X_{3}-0.01521X_{2}^{2}-0.00738X_{3}^{2}$ (4)	$Y_{Y}=4.316+3.229X_{2}+1.375X_{3}-6.08X_{2}^{2}-2.95X_{3}^{2}$ (5)
3	$Y_{C*}=14.672+0.2139X_{1}+0.2947X_{4}+0.039X_{4}^{2}$ (6)	$Y_{C*}=13.204+2.139X_{1}-0.136X_{4}+0.0295X_{4}^{2}$ (7)
4	$Y_{h*}=73.29+0.2455X_{1}-0.2005X_{3}+0.1261X_{4}+0.0675X_{4}^{2}-0.499X_{1}X_{4}$ (8)	$Y_{h*}=60.18+31.3X_{1}-4.01X_{3}+1.599X_{4}+0.051X_{4}^{2}-4.34X_{1}X_{4}$ (9)
5	$Y_{F}=4.1219+0.0682X_{1}-0.0305X_{2}+0.0113X_{3}-0.004X_{4}+0.0314X_{3}^{2}-0.242X_{1}X_{4}+0.267X_{2}X_{3}+0.325X_{2}X_{4}-0.391X_{3}X_{4}$ (10)	$Y_{F}=2.46+14.67X_{1}-64.9X_{2}+12.5X_{3}+1.336X_{4}+12.56X_{3}^{2}-2.103X_{1}X_{4}+106.9X_{2}X_{3}+5.66X_{2}X_{4}-6.81X_{3}X_{4}$ (11)

^1^ X_1_, X_2_ and X_3_ factors are presented in Table 1.

## Data Availability

Not applicable.

## Supplementary Materials

The following are available online at https://www.mdpi.com/2304-8158/10/2/392/s1, Table S1: Values of quality indices for oils EVOO and ROO, used as starting materials. Figure S1: GC-MS chromatographic profiles of oregano, lemon balm and bay laurel EOs. Mass spectra of peaks were cross-referenced against the NIST mass spectral library (Version 2.0f, 2008). Peak numbering according to Table S2 for each EO. Table S2: GC-MS data for the constituents of oregano, lemon balm and bay laurel EOs. Mass spectra of peaks were cross-referenced against the NIST mass spectral library (Version 2.0f, 2008). Figure S2: UV spectra at T0 (oil base), T1, T3, T6 and T12 of storage period of: EVOO oils (A); and CCD oils (B). An oil dilution factor of 2 was applied for spectra acquisition of CCD oils as compared to EVOO oils. Figure S3: Scatterplots (PC1 vs. PC2 and PC2 vs. PC3) of principle component analysis (PCA) for FT-MIR spectra of virgin olive oils: VOOs spectral database of the LFCT (dark green); flavored VOOs spectral data for CCD Samples 1–31 (yellow); base EVOO spectral data at storage time 0, 1, 6, 12 and 18 months (light green) (Samples 32–36). Spectra were obtained as described in Section 2.7.
