# Supplementary material for: Flavored Olive Oil as a Preservation Means of Reduced Salt Spanish Style Green Table Olives (cv. Chalkidiki)
Source: Foods. 2021 Feb 11;10(2):392. doi: 10.3390/foods10020392 (PMC7916839; doi:10.3390/foods10020392)
Supplement: Supplementary file 1 [file foods-10-00392-s001.pdf]

Flavored olive oil as a preservation means of reduced salt Spanish style green table olives (cv. Chalkidiki)

Maria Papapostolou[1], Fani Th. Mantzouridou[1,2] and Maria Z. Tsimidou[1,2]*

[1] Laboratory of Food Chemistry and Technology (LFCT), School of Chemistry, Aristotle University of Thessaloniki (AUTh), 54124 Thessaloniki, Greece; papaposm@chem.auth.gr (M.P.); fmantz@chem.auth.gr (F.Th.M.)

[2] Natural Products Research Center of Excellence (NatPro-AUTH), Center for Interdisciplinary Research and Innovation (CIRI-AUTH), 57001 Thessaloniki, Greece

* Correspondence: tsimidou@chem.auth.gr (M.Z.T); Tel: +302310997796

Supplementary materials

Table S1. Values of quality indices for oils EVOO and ROO, used as starting materials.

Quality index	EVOO	Upper limits EVOO [1]	ROO	Upper limits ROO [1]
Acidity (% oleic acid)	0.6 ± 0.0	≤ 0.8	0.3 ± 0.0	≤ 0.30
PV (meq O_2/kg oil)	10.7 ± 0.5	≤ 20	4.9 ± 0.1	≤ 5.0
K_{232}	2.0 ± 0.0	≤ 2.5	-	-
K_{270}	0.1 ± 0.0	≤ 0.22	0.94± 0.0	≤ 1.25
Total Htyr & Tyr (mg/20g oil)	8.3 ± 0.0	-	0.0	-

[1] EU 2568/91 and amendments

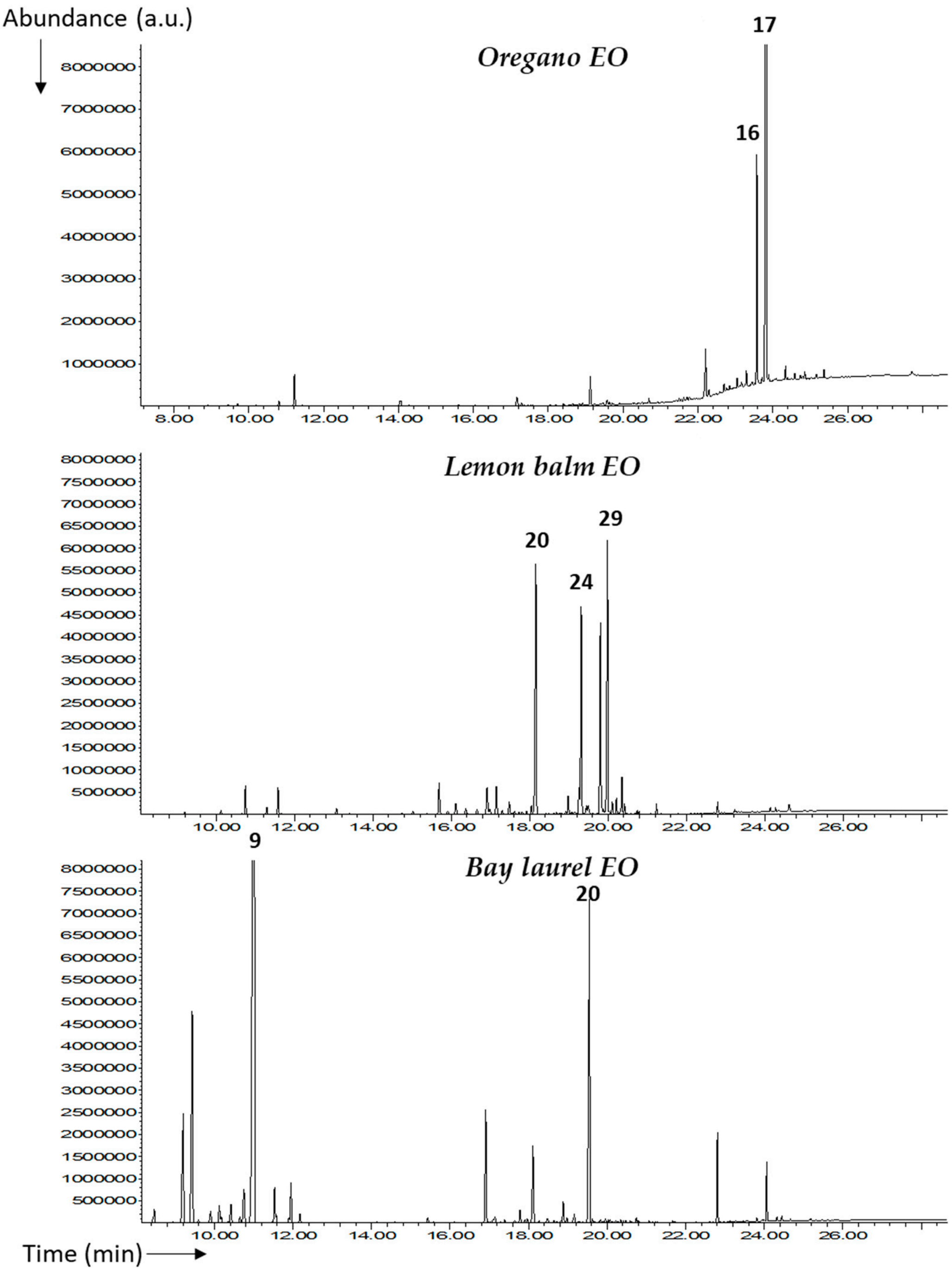

Figure S1. GC-MS chromatographic profiles of oregano, lemon balm and bay laurel EOs. Mass spectra of peaks were cross-referenced against the NIST mass spectral library (Version 2.0f, 2008). Peak numbering according to Table S2 for each EO.

Table S2. GC-MS data for the constituents of oregano, lemon balm and bay laurel EOs. Mass spectra of peaks were cross-referenced against the NIST mass spectral library (Version 2.0f, 2008).

Peak No	RT*	Compound	Abundance %	Peak No	RT*	Compound	Abundance %
			**Oregano EO**				
**1**	9.72	(+)-2-carene	0.13	**10**	19.58	unidentified	0.24

**2**	10.81	γ-terpinene	0.24	**11**	19.64	2-isopropyl-5-methyl-9-methylene-bicyclo-1-decene(4.4.0)	0.24
**3**	11.23	*p*-cymene	1.46	**12**	20.51	(-)-calamenene	0.16
**4**	14.06	dehydro-*p*-cymene	0.55	**13**	20.69	unidentified	0.27
**5**	17.17	α-caryophyllene	0.56	**14**	22.20	caryophyllene oxide	2.73
**6**	17.29	terpinen-4-ol	0.18	**15**	23.05	*p*-cymen-7-ol	2.51
**7**	18.92	(-)-borneol	0.28	**16**	23.57	**thymol**	**10.57**
**8**	19.13	β-bisabolene	1.46	**17**	23.82	**carvacrol**	**78.17**
**9**	19.46	1.1.6-trimethyl-1.2-dihydronaphthalene	0.25			**Total**	**100**
			**Lemon balm EO**				
**1**	10.13	β-myrcene	0.24	**21**	18.92	aromadendrene	0.20
**2**	10.75	limonene	1.97	**22**	18.99	(*E*)-β-famesene	1.30
**3**	11.30	*trans*-β-ocimene	0.48	**23**	19.26	unidentified	1.81
**4**	11.58	*cis*-β-ocimene	1.75	**24**	19.32	**neral**	**14.41**
**5**	13.08	sulcatone	0.44	**25**	19.45	*cis*-geranic acid methyl ester	0.68
**6**	15.03	1-octen-3-ol	0.27	**26**	19.49	α-amorphene	0.80
**7**	15.69	citronellal	2.62	**27**	19.80	germacrene D	13.54
**8**	15.91	unidentified	0.29	**28**	19.89	2-isopropyl-5-methyl-9-methylene-bicyclo-1-decene(4.4.0)	0.29
**9**	16.12	α-copaene	0.98	**29**	19.99	**geranial**	**19.69**
**10**	16.37	unidentified	0.37	**30**	20.11	unidentified	0.88
**11**	16.66	(-)-β-bourbonene	0.40	**31**	20.22	2,6-octadien-1-ol, 3,7-dimethyl-, acetate	0.98
**12**	16.92	α-caryophyllene	2.48	**32**	20.35	δ-cadinene	2.55
**13**	16.99	β-cubebene	0.56	**33**	20.43	γ-cadinene	0.61
**14**	17.16	bicyclo[2.2.1]hept-2-ene, 2,7,7-trimethyl-	2.19	**34**	20.75	nerol	0.23
**15**	17.30	unidentified	0.30	**35**	20.79	α-muurolene	0.20
**16**	17.48	unidentified	1.20	**36**	21.24	*trans*-geraniol	0.55
**17**	17.62	α-santalene	0.30	**37**	22.80	caryophyllene oxide	0.73
**18**	17.92	(-)-β-elemene	0.25	**38**	24.63	τ-muurolol	0.71
**19**	18.05	unidentified	0.66			**Total**	**99.53**
**20**	18.16	**β-caryophyllene**	**21.62**				
			**Bay laurel EO**				
**1**	8.48	camphene	0.48	**14**	16.92	linalool	3.35
**2**	9.21	β-pinene	3.77	**15**	17.79	bornyl acetate	0.52
**3**	9.44	sabinene	6.51	**16**	17.92	β-elemene	0.09
**4**	9.92	(+)-3-carene	3.42	**17**	18.12	(-)-terpinen-4-ol	2.32
**5**	10.13	β-myrcene	0.53	**18**	18.88	*p*-mentha-1(7),8-diene	0.24

**6**	10.18	α-phellandrene	0.25	**19**	19.14	estragole	0.44
**7**	10.43	(+)-2-carene	0.45	**20**	19.55	**terpinyl acetate**	**14.27**
**8**	10.77	limonene	3.68	**21**	20.75	nerol	0.44
**9**	11.03	**eucalyptol**	**45.96**	**22**	22.81	methyl eugenol	4.54
**10**	11.55	γ-terpinene	1.14	**23**	23.82	spathulenol	0.08
**11**	11.96	*p*-cymene	3.89	**24**	24.07	eugenol	1.36
**12**	12.19	unidentified	0.40			**Total**	**98.32**
**13**	15.44	unidentified	0.19				

*Retention times (min) were measured from the injection point; major compounds are shown in bold.

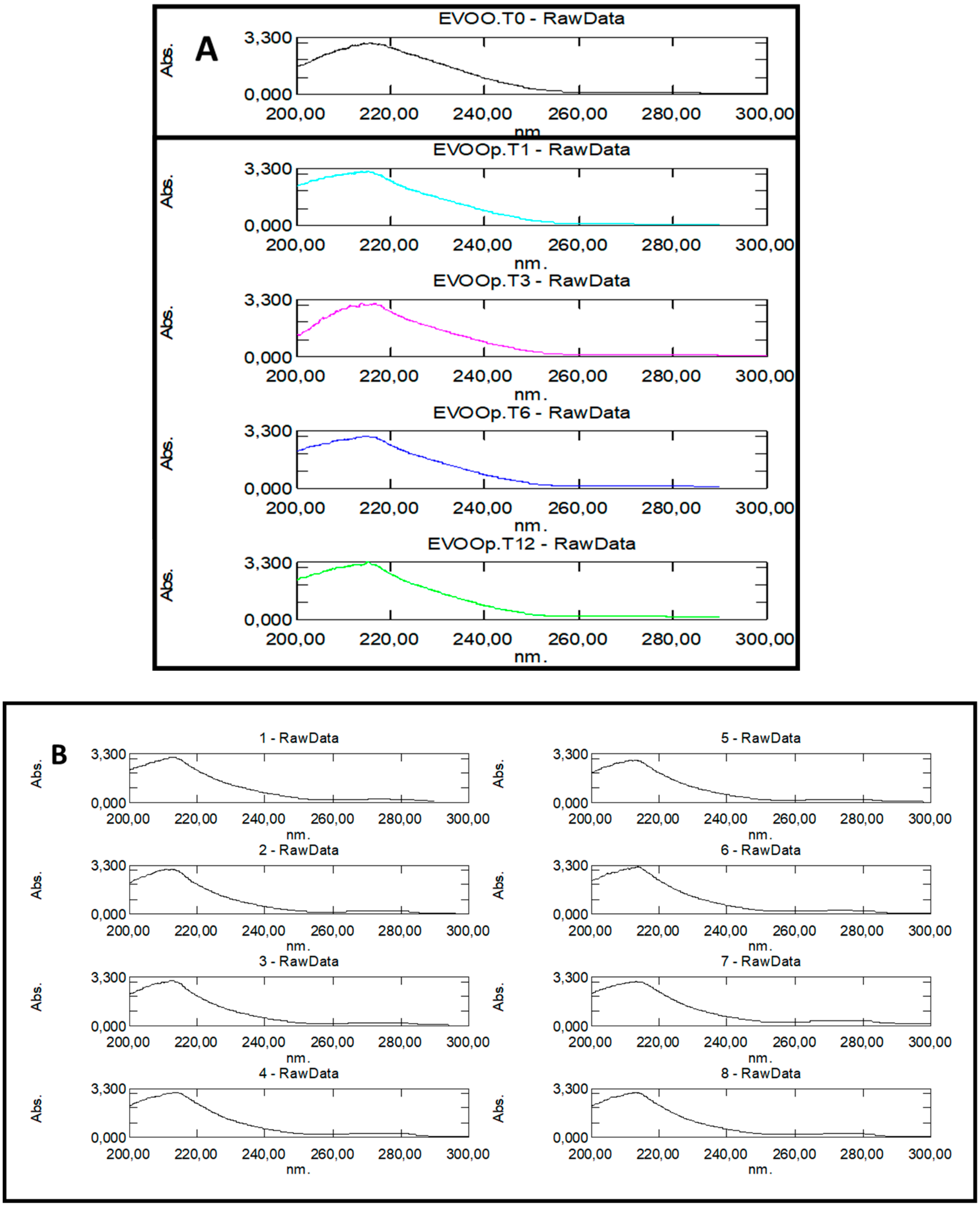

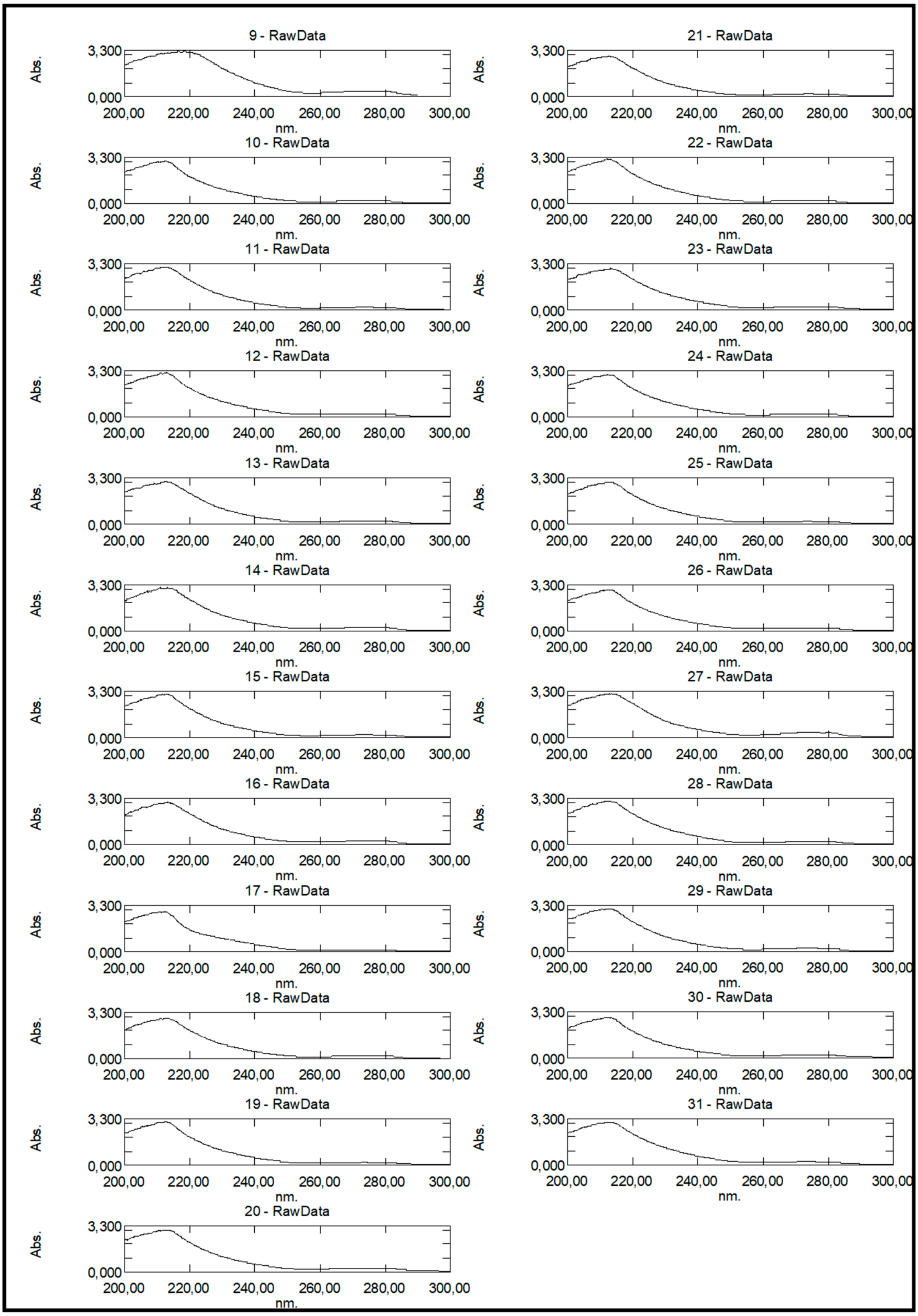

Figure S2. UV spectra of EVOO oils at T0 (oil base), T1, T3, T6 and T12 of storage period (A) and CCD oils (B). An oil dilution factor of 2 was applied for spectra acquisition of CCD oils as compared to EVOO oils.

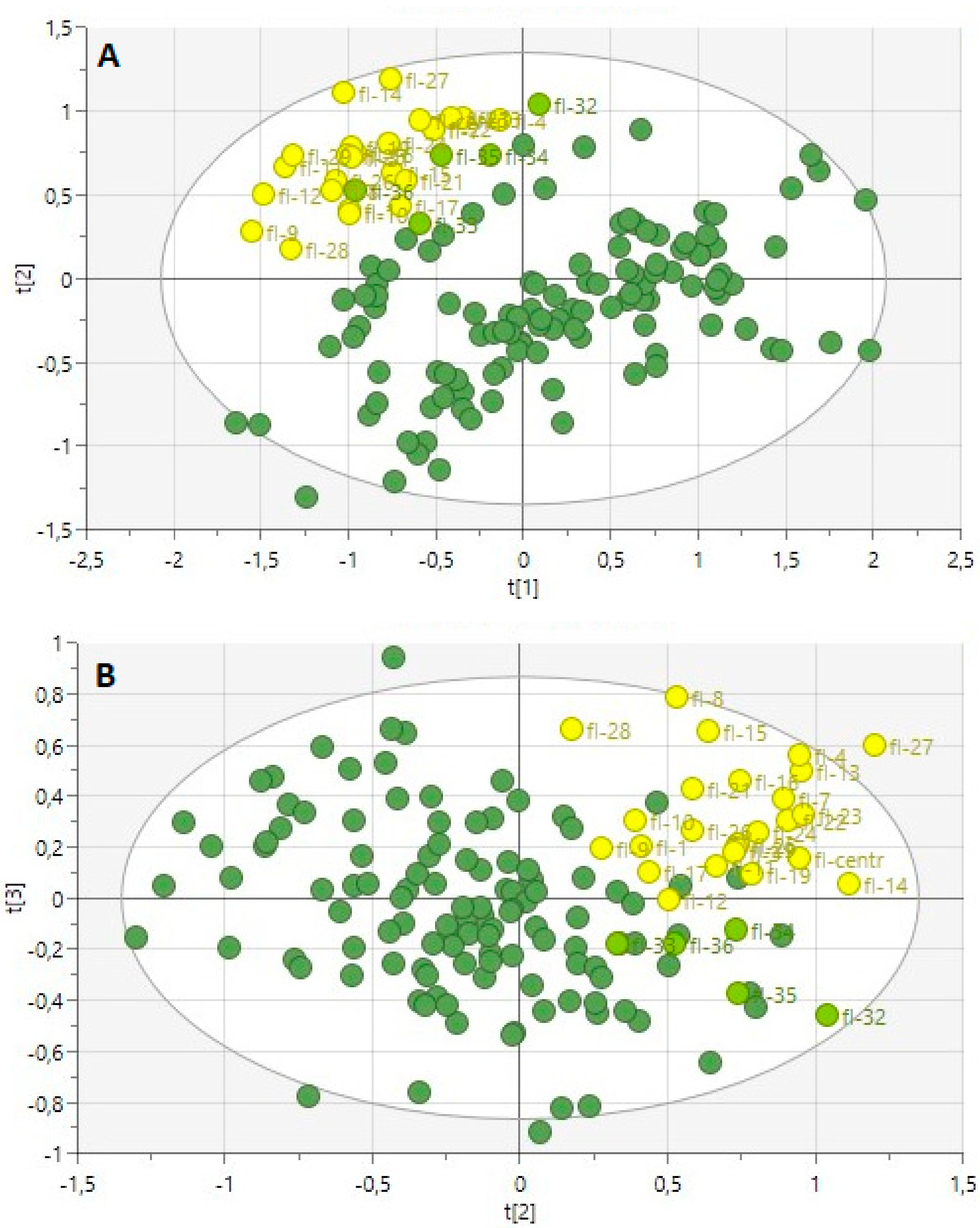

Figure S3. Scatterplots (PC1 vs PC2 and PC2 vs PC3) of principle component analysis (PCA) for FT-MIR spectra of virgin olive oils: VOOs spectral data base of the LFCT, (dark green); flavored VOOs spectral data for CCD samples 1-31 (yellow); base EVOO spectral data at storage time 0, 1, 6, 12, 18 months, light green (no 32-36). Spectra were obtained as described in § 2.7.
